# Supplementary material for: A Comparative Multianalytical Approach to the Characterization of Different Grades of Matcha Tea (Camellia sinensis (L.) Kuntze)
Source: Plants (Basel). 2025 May 27;14(11):1631. doi: 10.3390/plants14111631 (PMC12157965; doi:10.3390/plants14111631)
Supplement: Supplementary file 1 [file plants-14-01631-s001.zip › plants-3635553-supplementary.pdf]

plants

Supplementary Materials

# A Comparative Multianalytical Approach to the Characterization of Different Grades of Matcha Tea (*Camellia sinensis* (L.) Kuntze)

**Chiara Toniolo[1], Adriano Patriarca[2], Daniela De Vita[1], Luca Santi[1*] and Fabio Sciubba[1,3,4]**

[1] Department of Environmental Biology Sapienza University of Rome, Piazzale Aldo Moro, 5 – 00185 Rome Italy; chiara.toniolo@uniroma1.it (C.T.), daniela.devita@uniroma1.it (D.D.V.), l.santi@uniroma1.it (L.S.), fabio.sciubba@uniroma1.it (F.S.)
[2] Department of Chemistry, University of Rome Sapienza, Piazzale Aldo Moro 5, 00185 Rome, Italy; adriano.patriarca@uniroma1.it (A.P.)
[3] NMR-Based Metabolomics Laboratory (NMLab), Sapienza University of Rome, Piazzale Aldo Moro 5, 00185 Rome, Italy
[4] Interdepartmental Center of Applied Sciences for the protection of the environment and cultural heritage (CIABC), Sapienza University of Rome, Piazzale Aldo Moro 5, 00185 Rome, Italy
* Correspondence: l.santi@uniroma1.it; Tel. +39 0649912518

**List of Reagents, Solvents, and Standards**

A complete list of all chemical reagents and standards used is provided here for reference.

Standards and solvents were purchased from Sigma (Sigma-Aldrich, USA). All chemicals and solvents were of analytical grade. The stationary phase used for HPTLC analysis consisted of silica gel 60 F254 precoated plates (20 × 10 cm), purchased from Merck (USA).

Solvents (analytical grade) used for sample and standard preparation, as well as for mobile phases in HPTLC analysis, included: ethyl acetate, toluene, dichloromethane, chloroform, methanol, 1-butanol, acetone, acetic acid, and formic acid.

Chemicals and reagents for NMR analysis: deuterium oxide ($D_2O$), 3-(trimethylsilyl)-propionic-2,2,3,3-d4 acid sodium salt (TSP), deuterated chloroform ($CDCl_3$) hexamethyldisiloxane (HMDSO).

HPTLC standards included the following:

- Amino acids: alanine, arginine, asparagine, aspartic acid, cysteine, glutamic acid, glutamine, glycine, histidine, isoleucine, leucine, lysine, methionine, phenylalanine, proline, serine, theanine, threonine, tryptophan, tyrosine, and valine.
- Xanthine alkaloids: caffeine, theobromine, and theophylline.
- Organic acids and flavonoids: 3,5-di-caffeoylquinic acid, apigenin, caffeic acid, chlorogenic acid, cinnamic acid, gallic acid, hyperoside, kaempferol, luteolin, luteolin 7-*O*-glucoside, protocatechuic acid, quercetin, rutin, and shikimic acid.
- Catechins: catechin, epicatechin, catechin gallate, epicatechin gallate, epigallocatechin, and epigallocatechin gallate.

HPTLC derivatization agents included:

- For anisaldehyde-sulfuric acid reagent: anisaldehyde, sulfuric acid, acetic acid, and methanol.
- For Natural Product Reagent: phenylboronic acid and ethyl acetate.
- For ninhydrin reagent: ninhydrin, isopropanol, and acetic acid.

 https://doi.org/10.3390/plants14111631

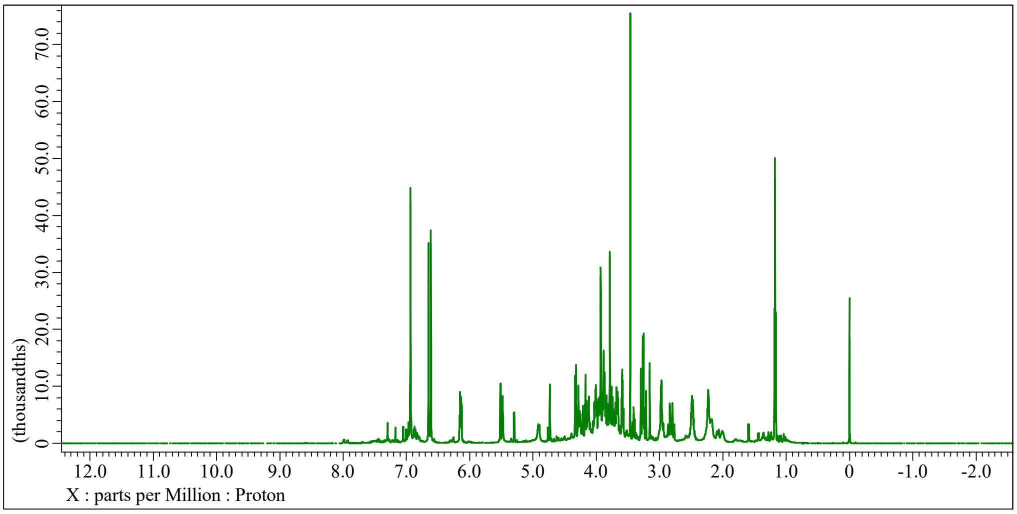


**Figure S1**. Hydroalcoholic extract full spectra of matcha tea sample.

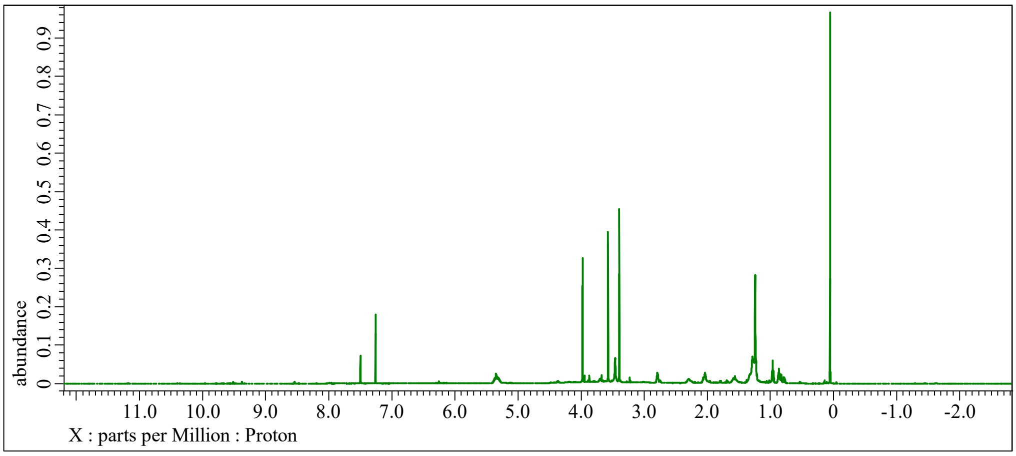


**Figure S2**. Organic extract full spectra of matcha tea sample.

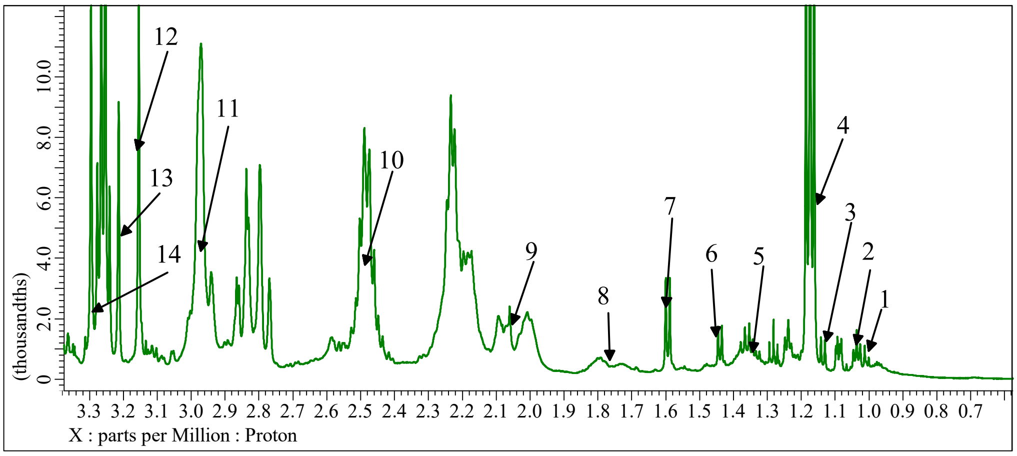


**Figure S3**. Hydroalcoholic extract spectra of matcha tea sample, region 0.7-3.3 ppm. 1. isoleucine, 2. leucine, 3. valine, 4. theanine, 5. fucose, 6. threonine, 7. alanine, 8. lysine, 9. quinic acid, 10. glutamine, 11. methylguanidine, 12. dimethylglycine, 13. choline, 14. betaine.

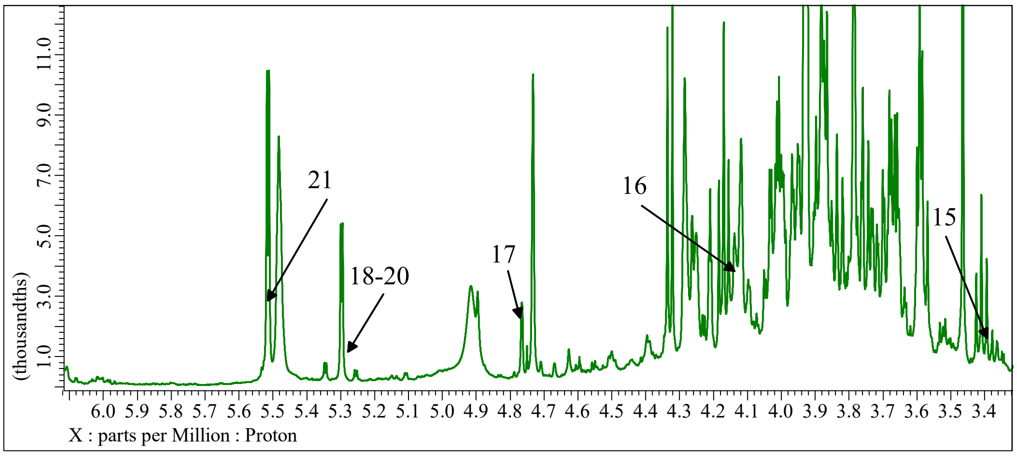


**Figure S4**. Hydroalcoholic extract spectra of matcha tea sample, region 3.4-6.0 ppm. 15. myo-inositol, 16. fructose, 17. ECG, 18. U01, 19. trehalose, 20. U02, 21. sucrose.

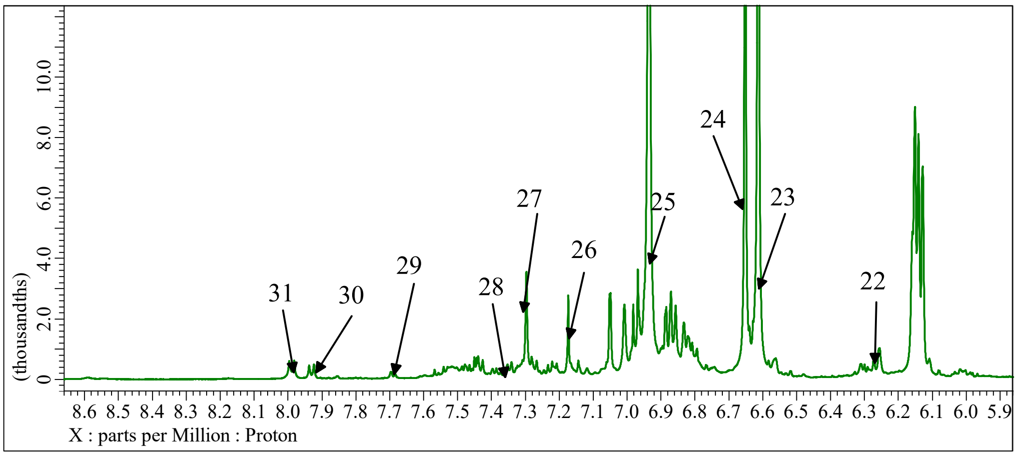


**Figure S5**. Hydroalcoholic extract spectra of matcha tea sample, region 5.9-8.6 ppm. 22. total chlorogenic acids, 23. EGCG, 24. EGC, 25. EC, 26. gallic acid quinic ester, 27. U03, 28. protocatechuic acid, 29. cinnamic acid quinic ester, 30. 4-hydroxybenzoic acid, 31. U04.

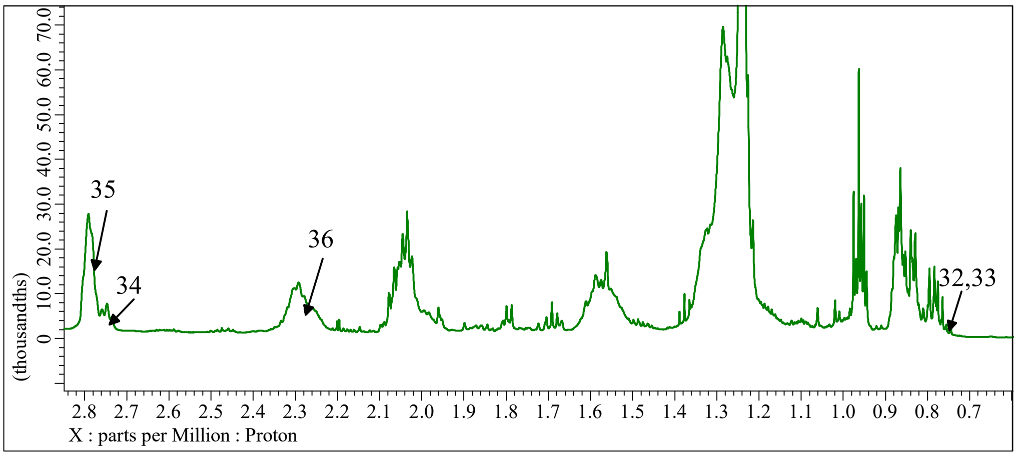


**Figure S6**. Organic extract spectra of matcha tea sample, region 0.7-2.8 ppm. 32. ergostenol+ergosterol, 33. cholestanol, 34. FA-omega 6, 35. FA-omega 3, 36. FA-omega 9.

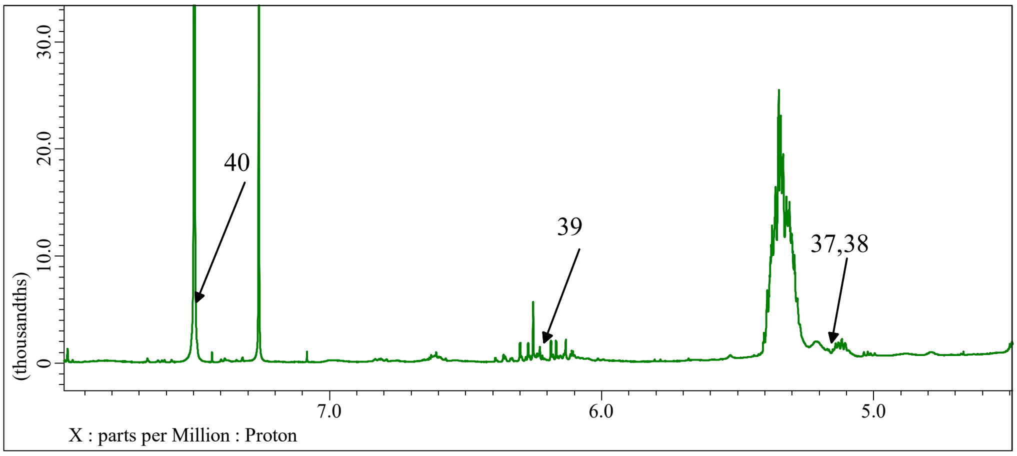


**Figure S7**. Organic extract spectra of matcha tea sample, region 5.0-7.6 ppm. 37. triacylglycerols, 38. glycerophospholipids, 39. α+β farnesene, 40. caffeine.

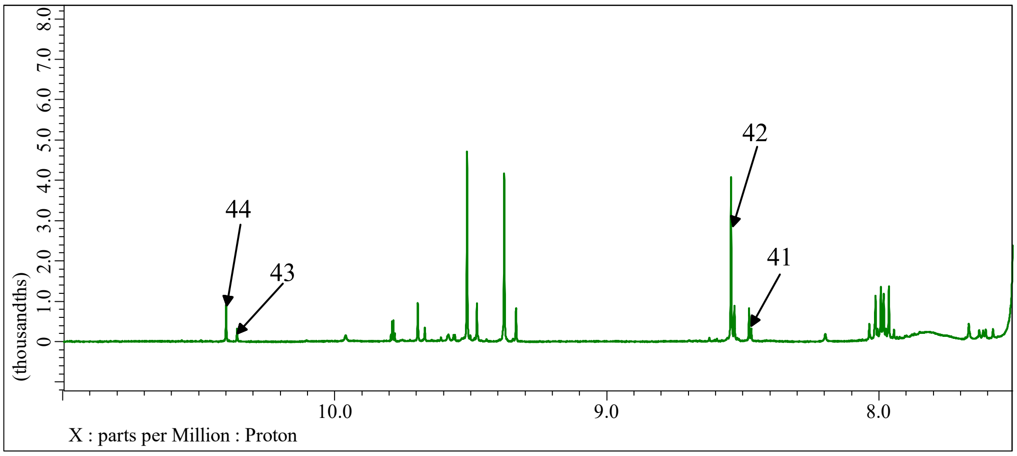


**Figure S8**. Organic extract spectra of matcha tea sample, region 8.0-11 ppm. 41. pheophytin A, 42. pyropheophorbide A, 43. pheophytin B, 44. pyropheophorbide B.

**Supporting Table S1.** Spectral assignation of each metabolite extracted from the $^{1}H$ spectrum of Matcha Tea

| Compound | $^{1}H$ δ ppm | Multiplicity | Assignment |
|---|---|---|---|
| **Amino acids** | | | |
| **Alanine** | **1.48**<br>3.80 | **d**<br>q | **β-CH₃**<br>α-CH |
| **DimethylGlycine** | 3.71<br>**2.91** | **s**<br>**s** | **CH**<br>**N-1,2 $CH_3$** |
| **Glutamine** | **2.14**<br>2.45<br>3.81 | **m**<br>m<br>m | **γ-$CH_2$**<br>β-$CH_2$<br>α-CH |
| **Isoleucine** | 0.95 | t | -$CH_3$ |

| | | | |
|---|---|---|---|
| | **1.02** | **d** | **γ-$CH_3$** |
| | 1.25 | m | γ′-CH |
| | 1.49 | m | γ′′-CH |
| | 1.99 | m | β-CH |
| | 3.69 | m | α-CH |
| **Leucine** | **0.97** | **m** | **δ,δ′-$CH_3$** |
| | 1.72 | m | γ-CH |
| | 1.73 | m | β-$CH_2$ |
| | 3.74 | m | α-CH |
| **Lysine** | 3.73 | t | α-CH |
| | 1.89 | m | -$CH_2$ |
| | 1.43 | m | -$CH_2$ |
| | **1.71** | **m** | **- $CH_2$** |
| | 3.03 | t | ε- $CH_2$ |
| **Theanine** | **1.17** | **t** | **$CH_3$** |
| | 3.26 | m | N-$CH_2$ |
| | 3.70 | m | α-CH |
| | 2.40 | m | -$CH_2$ |
| | 2.13 | m | -$CH_2$ |
| **Threonine** | **1.32** | **d** | **γ-$CH_3$** |
| | 3.60 | m | α-CH |
| | 4.27 | m | β-CH |
| **Valine** | 0.99 | d | γ-$CH_3$ |
| | **1.05** | **d** | **γ′-$CH_3$** |
| | 2.29 | m | β-CH |
| | 3.62 | m | α-CH |
| **Organic Acids** | | | |
| **4-Hydroxybenzoic acid** | **7.94** | **d** | **CH 2,6** |
| | 6.93 | d | **CH 3,5** |
| **Cinnamic acid quinic ester** | **6.50** | **d** | **CH 8** |
| | **7.49** | **m** | **CH 7,3,4** |
| | **7.69** | **m** | **CH 2,6** |
| **Total chlorogenic acids** | **6.30-6.44** | **d** | **CH8** |
| | 7.54-7.68 | d | CH7 |
| | 7.09 | dd | CH6 |
| | 7.20 | d | CH5 |
| | 6.96 | d | CH2 |
| **Gallic acid quinic ester** | **7.17** | **s** | **CH 2,6** |
| **Quinic acid** | 2.04; 1.97 | dd | 2,2′-$CH_2$ |
| | 2.08; 1.88 | dd | 6,6′-$CH_2$ |
| | **4.16** | **m** | **CH-3** |
| | 3.56 | m | CH-4 |
| | 4.03 | m | CH-5 |

| | | | |
|---|---|---|---|
| **Protocatechuic acid** | 7.33 | d | CH-1 |
| | **7.43** | dd | CH-6 |
| | 6.92 | d | CH-5 |
| **Polyols and Carbohydrates** | | | |
| **Fructose** | 3.69 | m | CH-1 |
| | \ | \ | C-2 |
| | 4.22 | d | CH-3 |
| | 4.06 | m | CH-4 |
| | 3.90 | m | CH-5 |
| | 3.82 | m | CH-6 |
| **Fucose** | **5.21** | **d** | CH-1 |
| | 4.55 | **m** | C-2 |
| | 4.20 | **m** | CH-3 |
| | 3.86 | **m** | CH-4 |
| | 3.79 | **m** | CH-5 |
| | 3.65 | **m** | CH-6 |
| | 1.22 | **d** | $CH_3$ |
| **Myo-Inositol** | 4.05 | dd | CH-1 |
| | 3.61 | m | CH-2,2′ |
| | 3.43 | m | CH-3,3′ |
| | **3.27** | **t** | **CH-4** |
| **Sucrose** | **5.44** | **d** | **G CH-1** |
| | 3.59 | m | CH-2 |
| | 3.79 | m | CH-3 |
| | 3.48 | m | CH-4 |
| | 3.85 | m | CH-5 |
| | 3.82 | m | $CH_2$-6 |
| | 3.69 | m | F $CH_2$-1′ |
| | \ | \ | C-2 |
| | 4.22 | m | CH-3′ |
| | 4.06 | m | CH-4′ |
| | 3.90 | m | CH-5′ |
| | 3.82 | m | $CH_2$-6 |
| **Trealose** | **5.20** | **d** | **CH-1** |
| | 3.64 | m | CH-2 |
| | 3.86 | m | CH-3 |
| | 3.46 | m | CH-4 |
| **Lipids & Sterols** | | | |
| **Saturated fatty acid (SFA)** | 0.87 | t | $CH_3$ |
| | 1.26 | m | n-$CH_2$ |
| | 1.62 | m | $C\underline{H}_2$-$CH_2$-$CO_2^-$ |
| | **2.30** | **t** | **$CH_2$-$CO_2^-$** |

| | | | |
|---|---|---|---|
| **Monounsaturated ω-9 fatty acid (ω-9 FA)** | 0.88 | t | $CH_3$ |
| | 1.27 | m | n-$CH_2$ |
| | **2.03** | **m** | **$CH_2$-CH=CH** |
| **Polyunsaturated ω-6 fatty acid (ω-6 FA)** | 0.86 | t | $CH_3$ |
| | 1.36 | m | n-$CH_2$ |
| | 2.04 | m | $C\underline{H}_2$-CH=CH |
| | 5.37 | m | CH=CH |
| | **2.76** | **t** | **=CH-$C\underline{H}_2$-CH=** |
| | 2.06 | m | $C\underline{H}_2$-$CH_2$-$CO_2^-$ |
| | 2.31 | t | $CH_2$-$CO_2^-$ |
| **Polyunsaturated ω-3 fatty acid (ω-3 FA)** | 0.95 | t | $CH_3$ |
| | 1.37 | m | n-$CH_2$ |
| | 2.04 | m | $C\underline{H}_2$-CH=CH |
| | 5.36 | m | CH=CH |
| | **2.82** | **t** | **=CH-$C\underline{H}_2$-CH=** |
| | 2.03 | m | $C\underline{H}_2$-$CH_2$-$CO_2^-$ |
| | 2.30 | t | $CH_2$-$CO_2^-$ |
| **Glycerophospholipids** | 3.65-3,55 | dd | $CH_2$ |
| | 4.05-4.15 | dd | $CH_2$ |
| | **5.21** | **m** | **CH** |
| **Ergostenol+Ergosterol** | **0.66** | **s** | **$CH_3$-18** |
| | 5.55 | m | CH=6 |
| | 5.43 | m | CH=7 |
| | 5.27 | m | CH=22,23 |
| **TriacylGlycerols** | **5.25** | **m** | **CH** |
| | 4.15-4.29 | dd | 2$CH_2$ |
| **Other Metabolites** | | | |
| **Betaine** | **3.26** | **s** | **N-$(CH_3)_3$** |
| | 3.84 | s | $CH_2$ |
| **Caffeine** | **7.85** | **s** | **CH** |
| **Choline** | **3.21** | **s** | **N-$(CH_3)_3$** |
| | 3.51 | t | $CH_2$ |
| | 4.07 | t | $CH_2$ |
| **α+β Farnesene** | **6.35** | **m** | =$CH_2$ |
| | 6.60 | m | =CH |
| | 7.30 | dd | =CH |
| **Epicatechine (EC)** | **4.75** | **s** | |
| | **4.29** | | |

| | | | |
|---|---|---|---|
| **Epicatechinegallate (ECG)** | **6.94**<br>**6.83**<br>**7.02** | **s** | **CH 2,6** |
| **Epigallocatechine (EGC)** | **6.60**<br>**4.74** | **s**<br>**s** | **CH 2,6** |
| **Epigallocatechinegallate (EGCG)** | **6.62** | **s** | **CH 2,6** |
| **Methylguanidine** | **2.82** | **s** | **N-** $CH_3$ |
| **Pyropheophorbide A** | 6.18<br>6.28<br>8.01<br>**9.51** | dd<br>dd<br>m<br>**s** | |
| **Pyropheophorbide B** | 6.17<br>6.28<br>8.00<br>**10.04** | dd<br>dd<br>m<br>**s** | |
| **Pheophytin A** | **9.47** | **s** | |
| **Pheophytin B** | **10.35** | **s** | |
| **U01 (Myo-Inositol Glycosilate a)** | **5.25**<br>4.08 | **d**<br>m | |
| **U02 (Myo-Inositol Glycosilate b)** | 3.65<br>3.83<br>**5.34** | m<br>m<br>**d** | |
| **U03 (Caffeoyl Quinic Acid)** | **7.30** | **s** | **CH 2,6** |
| **U04(Uridine)** | **7.98**<br>6.95 | **d**<br>d | **1-CH** |

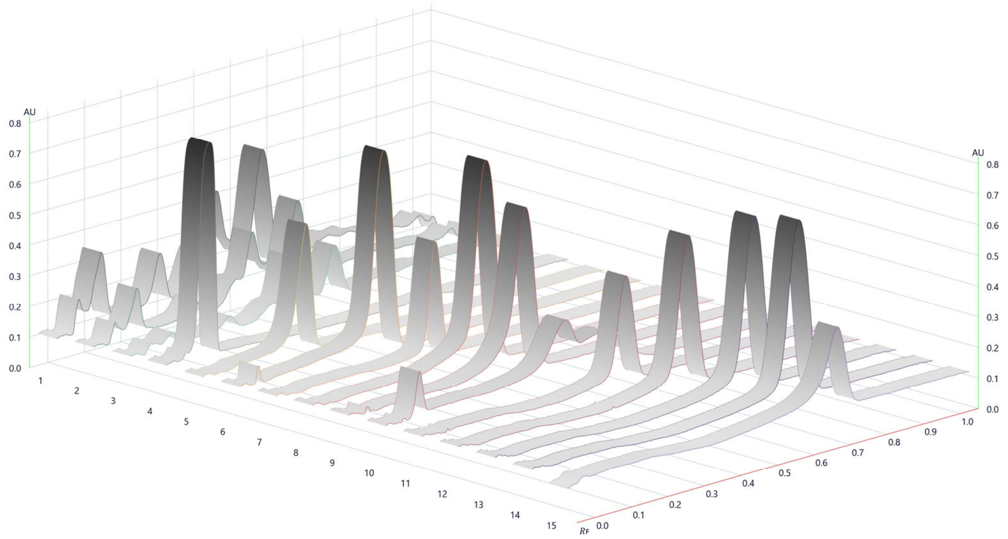

**Figure S9.** Densitometric scanning of HPTLC fingerprints of amino acids at 480 nm after derivatization with ninhydrine reagents. Track assignments: 1 hydroalcoholic extract of G1, 2 hydroalcoholic extract of G4, 3 hydroalcoholic extract of FG, 4 lysine, 5 arginine, 6 serine, 7 glutamic acid, 8 alanine, 9 threonine, 10 cysteine, 11 theanine, 12 valine, 13 methionine, 14 isoleucine, 15 leucine, 16 tyrosine.

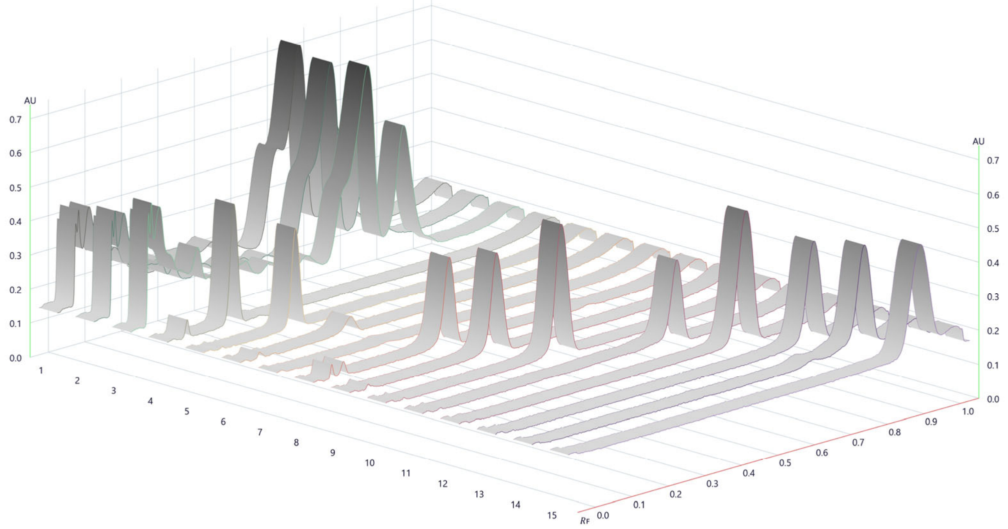

**(a)**

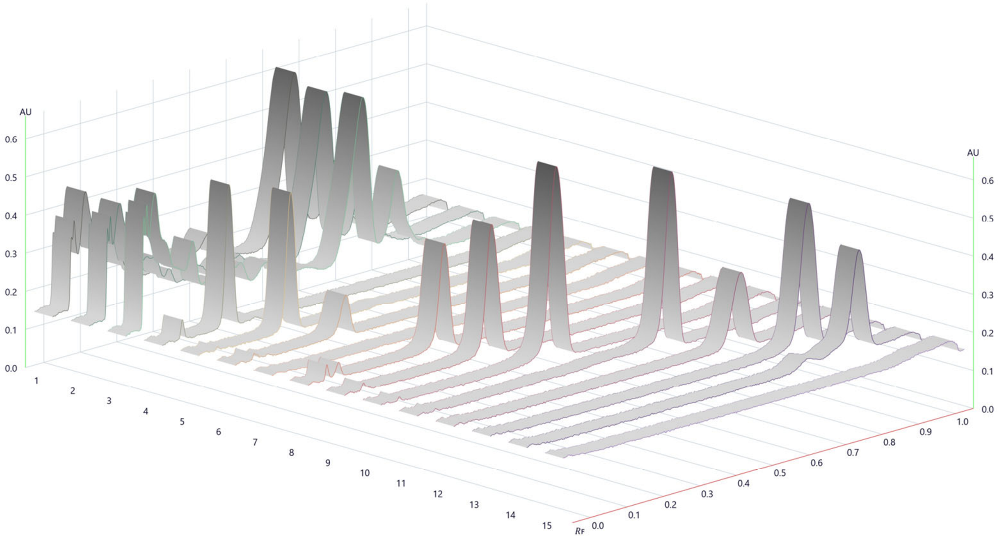


**(b)**

**Figure S10.** Densitometric scanning of HPTLC fingerprints of flavonoids and organic acids (a) at 272nm (b) 303. Track assignments: 1 hydroalcoholic extract of G1, 2 hydroalcoholic extract of G4, 3 hydroalcoholic extract of FG, 4 rutin, 5 kaempferol, 6 chlorogenic acid, 7 shikimic acid, 8 hyperoside, 9 luteolin 7-*O*-glucoside, 10 apigenin, 11 3,5-Di-caffeoylquinic acid, 12 gallic acid, 13 caffeic acid, 14 quercetin, 15 cinnamic acid.

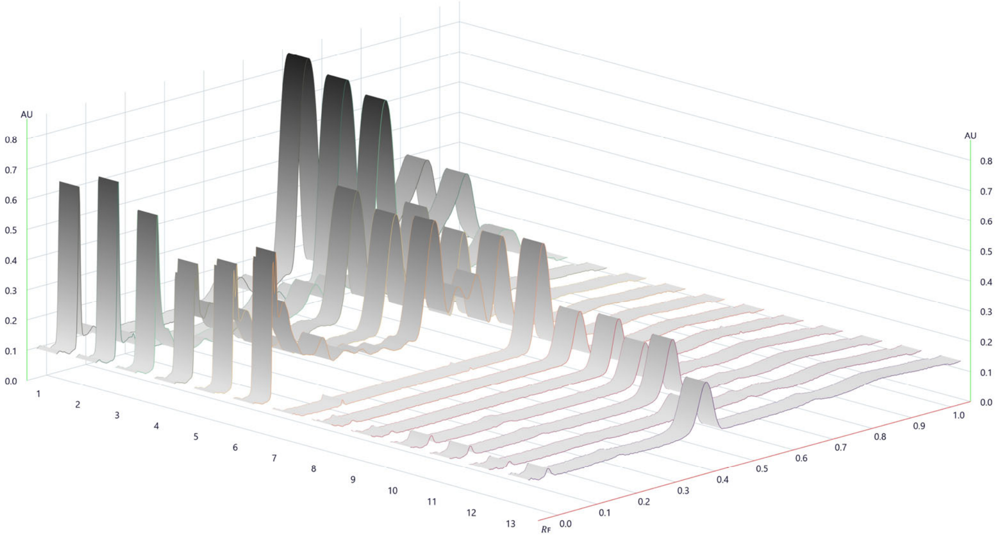


**Figure S11.** Densitometric scanning of HPTLC fingerprints of polyphenols at 272 nm. Track assignments: 1 organic extract of G1, 2 organic extract of G4, 3 organic extract of FG, 4 hydroalcoholic extract of G1, 5 hydroalcoholic extract of G4, 6 hydroalcoholic extract of FG, 7 caffeine, 8 catechin, 9 epicatechin, 10 catechin gallate, 11 epicatechin gallate, 12 epigallocatechin, 13 epigallocatechin gallate

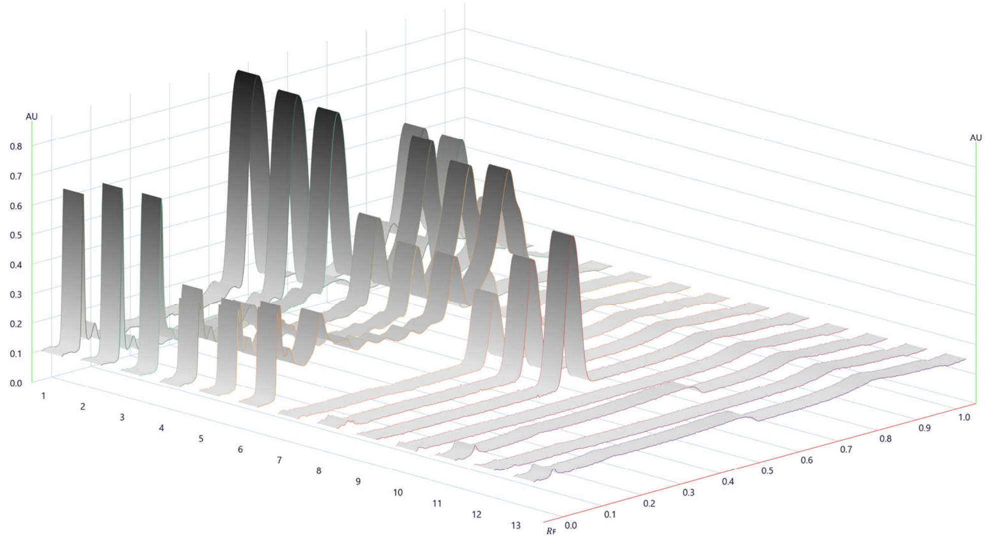


**Figure S12.** Densitometric scanning of HPTLC fingerprints of alkaloids at 272 nm. Track assignments: 1 organic extract of G1, 2 organic extract of G4, 3 organic extract of FG, 4 hydroalcoholic extract of G1, 5 hydroalcoholic extract of G4, 6 hydroalcoholic extract of FG, 7-9 caffeine (0,5; 1; 2 µg), 10 epicatechin, 11 epicatechin gallate, 12 epigallocatechin, 13 epigallocatechin gallate.

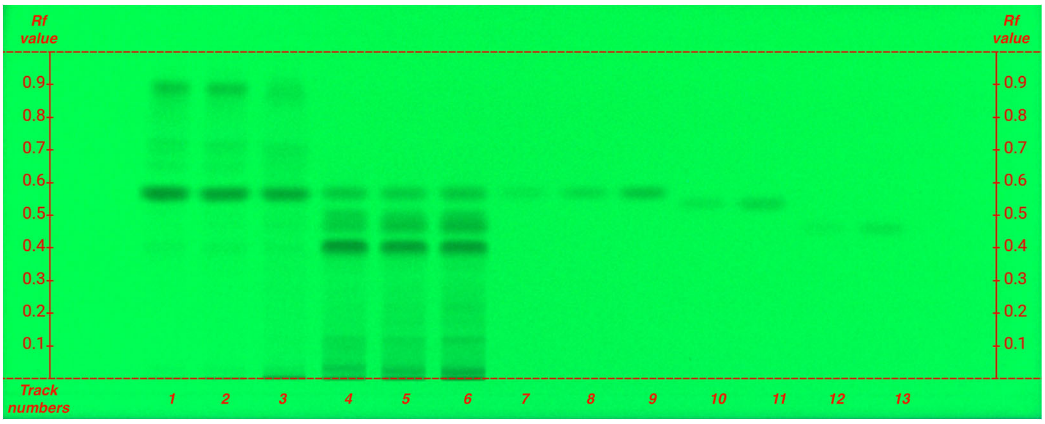


**Figure S13.** HPTLC fingerprints of alkaloids from both organic and hydroalcoholic phases of matcha tea samples, visualized under UV 254 nm without derivatization. Mobile phase: ethyl acetate/methanol/water (20:2.7:2 v/v). Track assignments: 1 organic phase of G1, 2 organic phase of G4, 3 organic phase of FG, 4 hydroalcoholic phase of G1, 5 hydroalcoholic phase of G4, 6 hydroalcoholic phase of FG, 7-9 caffeine (0,5; 1; 2 µg), 10-11 theophylline (0,5; 1 µg), 12-13 theobromine (0,5; 1 µg). The numbers ranging from 0.1 to 0.9 on the left and right sides of the image refer to the Rf values.

Although the images may suggest the presence of theobromine, the UV spectrum of the spot differs between the samples and the standard, indicating that the compound detected is likely not theobromine, but corresponds to epigallocatechin.
